# The Skin and Its Appendages, with Special Reference to the Development of the Teeth

**Published:** 1892-04

**Authors:** H. M. Fletcher


### The Skin and its Appendages, with Special Reference to the Development of the Teeth.

BY H. M. FLETCHER, D.D.S., M.D.

Read before the Mississippi Valley Dental Association March 10, 1892.

Mark Twain showed a good deal of knowledge and displayed a high appreciation of the extreme sensitiveness of that wonderful organ the skin, when he said he had found the best place to have a boil, and that that place was on some other fellow.

Mark had evidently either read the Book of Job, or had boils himself; for is there a disease of any other organ of the body that can bring about so pitiable a condition as in the case of the good old man Job when he was smitten with sore boils from the sole of his foot to his crown?

The skin is truly a most wonderful organ in many ways. Through it we receive many of the impressions that enter the brain, and thus add to our experience and knowledge.

Extending over the whole surface of the body, it may be regarded as a covering for the protection of the deeper tissues.

It is flexible, pliable, and elastic.

And loosely attached to the tissues and the organs which it covers, so that it slides easily over them allowing them ready play beneath it. In many parts of the body it is underlaid with a cushion of serous or adipose tissue which contains fat, thus insuring a smoothness, plumpness, and beauty of outline for the physical form.

In the palms of the hands, the soles of the feet and the scalp, the skin is not so loosely attached however, but adheres firmly to the tissues beneath.

On the face and neck it is very loose, very flexible and pliable, as is readily seen in the manifold changes in facial expression, and in the free movements of the neck.

### NERVE AND BLOOD SUPPLY.

It is very copiously supplied with nerve and blood vessels, and in both cases the branches which lead to the surface are from the same nerve or vessel that supplies the deeper tissues, hence an explanation of how topical application can effect a deep-seated trouble. The use of counter-irritants on the gums, "for instance," in cases of inflammation about the roots of the teeth, often effect a happy change.

The same reasoning and plan of treatment can be counted upon in almost any part of the body, but more especially the joints.

The skin is the seat of one of the most important of the five special senses, the touch. In this respect we find its highest development on the tips of the fingers and the lips. It is surprising to what an extent the sense of touch can be cultivated. This is best illustrated in the case of blind persons.

Almost every one has heard of Laura Bridgman, who was deaf, dumb, and blind. She was the first person so afflicted who became thoroughly educated. The chief means of reaching her mind was through the sense of touch.

A recent example of a similar, but much more marked, character is that of Helen Kellar, of Tuscumbia, Ala.

The skin is also one of the most important excretory organs of the body, in certain senses the most important. See what it contains:

1. Tactile, or touch, corpuscles.
2. Sweat glands.
3. Sebaceous glands.
4. Papillæ of skin.
5. The hair.
6. The nails.

And it also gives origin to the teeth, and to the crystalline lens of the eye, and to the more important parts of the nerves of special sense.

The sweat glands are about 1-300 of an inch in diameter.

They are fewest in the region of the back and neck, where they number about 400 to the square inch.

In the palms and the soles there are about 3,000 to the square inch.

It is estimated that there are two and one-half millions of these sweat ducts in the skin of the whole body on an average sized man.

This system of glands and ducts constitutes the sewerage system of the surface of the body.

It is estimated that if this two and one-half million ducts were extended and placed end to end, they would constitute 50,000 feet, or over nine miles of drainage of the system through the skin.

One thirty-seventh of the carbonic acid of the body is also secreted through these glands.

The skin is also capable of performing the function of absorption. To such an extent is this true that life may be preserved, in case of serious disorders of the digestive system, by the absorption of nutriment and remedies through the skin. In this way a life may be prolonged sufficiently for the digestive system to recover and resume its work again, and thus a life be kept from going out.

In certain low forms of animal life there is no mouth or stomach, nourishment being taken wholly by absorption through the skin. This type is illustrated in the tape worm. In this case, however, the food is digested by the animal upon which the parasite lives, and is then absorbed and assimilated by the worm.

### TISSUES DEVELOPED FROM THE SKIN.

It does not seem so impossible that one might almost have "nerves of steel" when we know that the enamel of the teeth—the hardest of all animal tissues—is developed from the skin, and at the same time the softest of all animal tissue the brain and nerves are developed from the same source.

Just see what a variety of organs we have developed from the epithelium or that organ from which is devoloped the outer coat of the skin, and how widely they differ in appearance and form.

The enamel of the teeth and the brain.

The crystalline lens of the eye, callous of the heel and the palms.

The nerves of special sense and the nails.

The hair which may be cut at will, and that extremely sensitive organ, the pulps of the teeth. The latter, however, are developed from a deeper portion of the skin than the others, and lastly the important parts of the sense of hearing, smelling and tasting.

### ANATOMY.

The skin may be divided into three layers :

1. The upper or outer layer is the epithelium, cuticle, or epidermis, modifications of which form all kinds of hard, horny out-growths or appendages of both animals and plants.

2. The *rete mucosa*, or papillary layer, which contains the papillæ,--these papillæ are highly vascular and contain the touch corpuscles.

3. The *cutis vera*, or true skin, which is the foundation for the other two layers, and is composed largely of connective tissue, holding in its meshes the fat of the skin, the sweat glands, the hair follicles, and from which the pulps of the teeth are developed.

The three layers are developed originally from the epiblast and mesoblast of the blastoderm.

Here a model of the blastoderma, with its three layers was displayed, and the manner of development of the different tissues from them explained.

SKIN OF PLANTS COMPARED WITH THAT OF ANIMALS.

Plants have a skin comparable to that of animals. Dr. Merrill Ricketts has compared the two as follows:

" Animals and plants alike have a circulatory and a respiratory system. Animals have a two-fold advantage over plants in that they have both the lung and external covering by which the act of respiration is carried on, while the plant has but the external covering (the skin). The skin of plants does not fill as many offices as does that of animals, yet there are many functions in common, viz.: that of absorption, protection, sensation, and of respiration.

"In addition to these, it serves in animals as an organ of excretion, perspiration, and the regulation of temperature.

" Their origin, structure and development, are practically the same, both having epithelium, from which the appendages of each develop.

" There is a correspondence from the shining coat of the polyp to the thick hide of the rhinoceros. Even the loose skin called the mouth, which envelopes the body of the mollusk, corresponds to the true skin of the higher animals, but we find it more highly developed in mammals. The dermis consists of a sheet of tough elastic tissue, composed of interlacing fibres, blood vessels, lymphatis, sweat glands, and nerves. It varies in thickness from the sixtieth of an inch to three or more inches; the thickest skins of both animals and plants being found in the tropics, a very singular fact indeed, especially when considered from a protective standpoint.

" Destroy either or all of the five senses; remove if you will a lung, a kidney, the spleen or pancreas; still we find that life can be sustained indefinitely.

" Remove a branch, a leaf, a flower or a fruit, we likewise find that life is sustained.

" How different it would be if we were to divest any animal or plant of its external covering. To remove or destroy one-fifth of the dermal covering of either, would cause almost instant death.

" By dermal covering I mean the skin or external membrane of either animals or plants. Its office in each is to protect, however it varies in structure.

" In the animal it is composed essentially of an internal layer formed principally of connective tissue, and rich in vessels and nerves called, the true skin or derma, and of an external layer composed of cells only, called the epidermis.

" It contains in addition many peculiar glandular and horny organs.

" In the plant it consists of epithelial tissue, husk and bark.

" Although they vary in structure there is a similarity. That found upon animals comprises that part of the skin which is raised in a blister and called the cuticle or scurf skin."

Many animals secrete from their skin such substances as form a means of protection for their bodies, or a house to live in, such as snails and other mollusks, the oyster and other shell-fish, and the many bright and beautiful coral.

The covering of the entire family of the crustaceans is a production of the skin, and serves the place of an internal bony skeleton such as is found in the vertebrata. This is seen in the coat of mail and the wings of insects.

The covering of all worms, whether it be the crust of the centipede, the slimy surface of the earth-worm, or the hairy coat of the caterpillar, all are variations of the epidermis.

Again, the scales of all fishes, the horny coat of the alligator, the shells of the tortoise and the armadillo, and the quills of the porcupine, are modifications of the skin.

One of the most beautiful productions of all nature is seen in the feathers of birds. Their varieties of beauty in form and color are almost endless. Feathers, together with beaks and claws of birds, are developed from this same source. The hair and the fur, the claws and the hoofs of animals, and the hair and the nails of human beings are appendages of this wonderful organ.

And last, but not least, among the appendages of the skin are the teeth of animals—including men—(excepting certain classes of fish), the tusks of elephants and walruses are simply enlarged or elongated teeth.

In the vegetable kingdom bark is another name for the skin, the leaves of trees and plants, and, conspicuous for their fragrance and beauty, the flowers of the vegetable kingdom, tanbark from the oak, corks from the bark of the cork-tree, canoes and other useful articles, from the bark of the birch tree,—these and others of the necessities and conveniences of life, are derived from the skin of vegetables.

"The thorn of the horny locust develops and falls off to give place to a new one, about the same process of development, or shedding, as takes place with the horns of deer and other animals. In the higher animals the epithelial scales are constantly being removed and replaced.

"In lizards and serpents the old epidermis is cast off entirely, being stripped from the head to the tail; in the toad it comes off in two pieces; in the frog in shreds; in fish and some mollusks in the form of slime.

"However modified, the epidermis or whatever its appendages, a like process of removal goes on. Animals shed their hair, horns and nails, birds their feathers, and crabs their shells.

"Desquamation, or falling off of epithelial cells, takes place throughout both kingdoms.

"Specimens of both animal and vegetable epidermis under the microscope reveal cellular structure, and their offices are the same. That portion of animal epidermis called the *rete mucosum* contains the nucleated cells and pigment or color granules on which the color of the animal depends."

The utility to mankind of the skins of animals and plants is a matter of experience and record from the remotest antiquity. Among barbarous and uncivilized races the skins of animals have, in large part, furnished their simple clothing, and at the same time their best protection from cold and from the constant dangers of injury from their rough surroundings. Teeth and horns of animals are used for weapons of defence and articles of adornment.

Indeed, in a sense, man's progress from barbarism to civilization is indicated largely by his skill in utilizing the pelts of animals and the fiber of the bark of plants and trees for clothing of his person and tents for his housing. Bark, fiber, wool and fur, are the materials out of which mankind first learned to make cloth, and there is no one present in this audience to-night but that is indebted to the skins of animals or plants for the clothing he or she wears. Our shoes are made of leather, and our clothing of wool, cotton, linen, or silk.

Here followed a stereoptican display of fifty lantern slides, mostly photomicrographs of the skin and its appendages, including sections of embryo chicks showing the mode of development of the epithelium.

